# Population and genome-wide association studies of *Sclerotinia sclerotiorum* isolates collected from diverse host plants throughout the United States

**DOI:** 10.3389/fmicb.2023.1251003

**Published:** 2023-09-27

**Authors:** Roshan Sharma Poudel, Kassaye Belay, Berlin Nelson, Robert Brueggeman, William Underwood

**Affiliations:** ^1^Department of Plant Pathology, North Dakota State University, Fargo, ND, United States; ^2^Department of Crop and Soil Sciences, Washington State University, Pullman, WA, United States; ^3^Edward T. Schafer Agricultural Research Center, Sunflower and Plant Biology Research Unit, USDA Agricultural Research Service, Fargo, ND, United States

**Keywords:** white mold, sunflower, stalk rot, stem rot, head rot, virulence factor

## Abstract

**Introduction:**

Sclerotinia sclerotiorum is a necrotrophic fungal pathogen causing disease and economic loss on numerous crop plants. This fungus has a broad host range and can infect over 400 plant species, including important oilseed crops such as soybean, canola, and sunflower. S. sclerotiorum isolates vary in aggressiveness of lesion formation on plant tissues. However, the genetic basis for this variation remains to be determined. The aims of this study were to evaluate a diverse collection of S. sclerotiorum isolates collected from numerous hosts and U.S. states for aggressiveness of stem lesion formation on sunflower, to evaluate the population characteristics, and to identify loci associated with isolate aggressiveness using genome-wide association mapping.

**Methods:**

A total of 219 S. sclerotiorum isolates were evaluated for stem lesion formation on two sunflower inbred lines and genotyped using genotyping-by-sequencing. DNA markers were used to assess population differentiation across hosts, regions, and climatic conditions and to perform a genome-wide association study of isolate aggressiveness.

**Results and discussion:**

We observed a broad range of aggressiveness for lesion formation on sunflower stems, and only a moderate correlation between aggressiveness on the two lines. Population genetic evaluations revealed differentiation between populations from warmer climate regions compared to cooler regions. Finally, a genome-wide association study of isolate aggressiveness identified three loci significantly associated with aggressiveness on sunflower. Functional characterization of candidate genes at these loci will likely improve our understanding of the virulence strategies used by this pathogen to cause disease on a wide array of agriculturally important host plants.

## Introduction

The ascomycete S*clerotinia sclerotiorum* (Lib.) de Bary is a necrotrophic fungal pathogen infecting over 400 plant sciences, including agriculturally important crops like sunflower, canola, and common bean (Boland and Hall, 1994; Bolton et al., 2006; Derbyshire et al., 2022). Diseases caused by *S. sclerotiorum* significantly impact sunflower production in the Northern Great Plains region of the United States, typically causing annual losses estimated at over 1% of total production and resulting in millions of dollars of economic losses (Heffer Link and Johnson, 2007; Markell et al., 2015; Harveson et al., 2016). Moreover, these losses can be much higher in years where weather conditions favor disease development. *S*. *sclerotiorum* can infect sunflower plants at any growth stage and causes three distinct diseases; basal stalk rot (also known as Sclerotinia wilt), mid-stalk rot, and head rot (Markell et al., 2015; Harveson et al., 2016). Basal stalk rot and head rot are persistent, economically significant diseases impacting U.S. sunflower production, while mid-stalk rot is observed sporadically and is generally regarded as less economically damaging (Markell et al., 2015; Harveson et al., 2016). Yield losses of 10%–20% due to head rot are typical in most years. However, in years where environmental conditions favor disease, losses of up to 80% have been reported (Harveson et al., 2016; Gulya et al., 2019). Additionally, head rot further impacts sunflower production by reducing seed oil content by 10% to 15% (Gulya et al., 2019). *S. sclerotiorum* produces resting structures referred to as sclerotia that contaminate soil and can remain viable for many years, complicating disease management practices (Bolton et al., 2006). Additionally, chemical control of basal stalk rot and head rot is generally ineffective and sunflower hybrids with complete resistance or even high levels of resistance to these diseases are not available (Qi et al., 2016; Seiler et al., 2017). Breeding for sunflower lines and hybrids with improved resistance is considered the most effective and economical avenue for controlling sunflower diseases caused by *S. sclerotiorum* (Qi et al., 2016). Progress is being made to identify diverse sources of resistance alleles and map genetic loci contributing to sunflower resistance against *S. sclerotiorum* (Davar et al., 2010; Amouzadeh et al., 2013; Talukder et al., 2016; Seiler et al., 2017; Zubrzycki et al., 2017; Filippi et al., 2020; Talukder et al., 2021, 2022a,b). However, resistance breeding alone may not be a durable solution. Historically, resistant varieties have suffered resistance breakdown, sometimes within a short period of time (Brown, 1995). Often, release of a novel resistant variety leads to its extensive monoculture, thereby exerting strong selection pressure on the local pathogen population (McDonald and Linde, 2002). Substantial selection pressure induces the pathogen to evolve to better adapt against resistant cultivars. Thus, understanding the evolutionary potential of a spreading pathogen population, its genetic structure, and genetic components involved in pathogenicity may help breeders design rational breeding schemes to achieve durable resistance (McDonald and Linde, 2002; Kamvar et al., 2017). Isolates of *S. sclerotiorum* have been reported to vary considerably in numerous traits related to growth and development as well as pathogenicity. This includes variation in rates of *in vitro* hyphal growth, pigmentation of mycelia, size and number of sclerotia formed, and aggressiveness in causing lesions on host plants (Otto-Hanson et al., 2011; Attanayake et al., 2012; Rather et al., 2022). Consequently, a comprehensive understanding of isolate diversity for this pathogen is important to facilitate resistance breeding efforts.

Over the past 20 years, several studies have applied population genetic approaches to evaluate the genetic diversity of *S. sclerotiorum* isolates collected from specific plant species or a specific location (Kohli et al., 1992; Cubeta et al., 1997; Atallah et al., 2004; Winton et al., 2006; Wu and Subbarao, 2006; Malvarez et al., 2007). Additionally, a small number of studies have evaluated more extensive isolate collections from multiple host and locations in the U.S. (Carbone and Kohn, 2001; Attanayake et al., 2014; Aldrich-Wolfe et al., 2015; Kamvar et al., 2017). Many of these genetic characterization studies employed a macroscopic assay to identify mycelial compatibility groups (MCGs) believed to represent groupings that identify clonal lineages or closely related isolates (Carbone et al., 1999; Aldrich-Wolfe et al., 2015). Various approaches and markers systems have been used to characterize genetic relatedness among *S. sclerotiorum* isolates including DNA fingerprinting (Cubeta et al., 1997; Malvarez et al., 2007), sequence analysis of rDNA [internal transcribed spacer (ITS) region] and protein-coding genes [elongation factor 1 alpha (EF-1α, 350 bp), calmodulin (CAL, 500 bp), chitin synthase 1 (CHS), actin (ACT,) and ras protein (RAS)] (Carbone and Kohn, 2001; Malvarez et al., 2007), and polymorphic microsatellite markers (Atallah et al., 2004; Attanayake et al., 2014; Aldrich-Wolfe et al., 2015; Kamvar et al., 2017). Several studies used both macroscopic and molecular marker assays to report the genetic diversity of *S. sclerotiorum* isolates (Attanayake et al., 2014; Aldrich-Wolfe et al., 2015; Kamvar et al., 2017). Although mycelial compatibility is genetically controlled, studies have shown conflicting evidence for the correlation between MCGs and neutral genetic markers (Schafer and Kohn, 2006; Aldrich-Wolfe et al., 2015; Kamvar et al., 2017). However, population genetic studies carried out by these independent research groups have improved understanding of the genetic diversity of *S. sclerotiorum* in the U.S. Aldrich-Wolfe et al. (2015) analyzed MCGs and microsatellite haplotypes of 145 pathogen isolates collected from multiple hosts to report a lack of host-specific barriers to gene flow for *S. sclerotiorum* across the North Central region of the U.S. Upon analyzing 156 *S. sclerotiorum* isolates collected from soybean grower fields and white mold screening nurseries across multiple U.S. states and four countries, Kamvar and colleagues recommended breeders to continue multi-site screening of new soybean varieties to account for genetic and phenotypic variation observed among *S. sclerotiorum* populations across the U.S. (Kamvar et al., 2017).

One limitation of past population studies focused on *S. sclerotiorum* is the use of relatively small numbers of molecular markers. Ideally, population genetic studies will utilize large numbers of diagnostic molecular markers distributed across the entire genome. Recent advancements in next-generation sequencing (NGS) and chromosome level reference genome assemblies have enhanced the accurate detection of single nucleotide polymorphisms (SNP), a type of frequently occurring genetic marker. Restriction enzyme-based genotyping-by-sequencing (GBS) has revolutionized the ability to obtain low-cost and high-density SNP markers from complex plant and pathogen species (Elshire et al., 2011; Poland et al., 2012; Leboldus et al., 2015). GBS utilizes restriction enzymes to target sequencing to a subset of the genome, thereby reducing genomic complexity and cost of sequencing. GBS has been successfully utilized in many genetic and genomic studies, including population and phylogenetic studies as well as genome-wide association mapping studies for plant-pathogenic fungi (Leboldus et al., 2015; Gao et al., 2016; Aoun et al., 2020).

The availability of a more extensive set of diagnostic SNPs generated by sequencing-based approaches also provides a resource for performing genome-wide association studies (GWAS) to map loci affecting a trait of interest. GWAS has been utilized extensively to study plant resistance to pathogens. Additionally, efforts are now being made to explore pathogenicity using GWAS with natural pathogen populations (Bartoli and Roux, 2017; Sánchez-Vallet et al., 2018; Shakouka et al., 2022). In this study, we utilized GBS to genotype a large and diverse collection of *S. sclerotiorum* isolates. Major objectives of the study were to: (1) evaluate aggressiveness of diverse *S. sclerotiorum* isolates in causing stem lesions on two sunflower inbred lines; (2) assess the population characteristics of a collection of *S. sclerotiorum* isolates from multiple host plants collected throughout the U.S. using SNP markers derived from GBS; and (3) conduct a genome-wide association study to identify loci associated with isolate aggressiveness on sunflower stems.

## Materials and methods

### *Sclerotinia sclerotiorum* isolates

A total of 219 *S. sclerotiorum* isolates were used in this study. Isolates were collected from 27 U.S. states and 25 host plants. Additionally, four isolates collected in Argentina and a single isolate each collected in Canada and Tasmania were included. Sampling was conducted by collecting sclerotia from diseased plant tissue. Isolates designated BN and JS were kindly provided by Dr. Berlin Nelson (Department of Plant Pathology, North Dakota State University, Fargo, ND) and Dr. James Steadman (Department of Plant Pathology, University of Nebraska, Lincoln, NE), respectively. Subsets of these isolates have been described in previous studies (Otto-Hanson et al., 2011; Aldrich-Wolfe et al., 2015). Detailed information for isolates used in this study is presented in Supplementary Table S1.

### Inoculum preparation

For all inoculum preparation, initial cultures of *S. sclerotiorum* isolates were produced by plating a single sclerotium on potato dextrose agar (PDA) followed by incubation at 22°C. To prepare inoculum for sunflower stem inoculations, initial PDA cultures were grown for 4 days and a 5 mm plug was subsequently excised from the edge of the growing colony using a cork borer and transferred mycelium-side down to a new plate of minimal media agar [25 mM NaOH, 22 mM DL-Malic Acid, 25 mM NH_4_NO_3_, 0.08 mM MgSO_4_, 39% agar (Guo and Stotz, 2007)]. Isolates were then grown on minimal media agar for 24 h at 22°C. Twelve 7 mm diameter circular sterile filter paper discs (Whatman #2) were subsequently placed around the growing edge of the colony to allow the fungus to colonize the discs and minimal media agar plates were incubated for an additional 24 h at 22°C. Colonized filter paper discs were used as inoculum for sunflower stem inoculations described below. *S. sclerotiorum-*infested millet seed inoculum for sunflower basal stalk rot inoculations was prepared as described previously (Underwood et al., 2021). Briefly, white proso millet seed (1.45 kg) was soaked for 16 h in 3 liters of water in metal trays, then strained. Three hundred milliliters of water were added, and trays were covered with foil and autoclaved twice with 25 min exposure times, stirring between autoclave cycles. After cooling, autoclaved millet was inoculated with PDA bearing mycelium of the selected *S. sclerotiorum* isolate by cutting up four 90 mm PDA plates of actively growing culture and stirring into the autoclaved millet. Inoculated millet was incubated for 3 days at 22°C, stirred under aseptic conditions, and incubated for an additional 3 days. Millet inoculum was then dried for 5 days at 30°C and stored at 4°C prior to use as inoculum for basal stalk rot inoculations described below.

### Sunflower stem lesion aggressiveness evaluations

*S. sclerotiorum* isolates were evaluated for aggressiveness in causing stem lesions on two sunflower inbred lines, HA 207 and HA 441 (Stafford and Thompson, 1983; Miller and Gulya, 2006). Inbred line HA 207 was developed for resistance to charcoal rot caused by *Macrophomina phaseolina* and development of this line involved a cross with a wild *Helianthus annuus* accession (Stafford and Thompson, 1983). Inbred line HA 441 was developed for improved resistance to Sclerotinia head rot (Miller and Gulya, 2006). For all evaluations, plants were sown in 24-position cell-packs (cell dimensions 5.7 cm × 7.6 cm) filled with potting mix (Sunshine SB 100B, SunGro Horticulture, Bellevue, WA, United States) and grown in a greenhouse at 22 ± 3°C with supplemental lighting to provide a 16 h photoperiod. Six-weeks-old plants were inoculated by placing a filter paper inoculum disc on the stem midway between the second and third internodes and affixing with laboratory film. Stem lesion lengths were measured using digital calipers at 8 days post-inoculation (dpi). Isolate evaluations on both inbred lines were carried out in α-lattice designs in which each isolate was inoculated onto 30 plants of each sunflower inbred line.

### Sunflower basal stalk rot disease evaluations

Comparisons of sunflower basal stalk rot disease caused by selected *S. sclerotiorum* isolates collected from cool or warm climates were conducted on moderately susceptible inbred line HA 89 and moderately resistant line RHA 801 (Roath et al., 1981; Underwood et al., 2021). Plants were sown in 32-position sheet pots (6.58 cm width × 6.58 cm length × 7.62 cm depth) and grown in the greenhouse for 5 weeks at 25 ± 3°C. Plants were inoculated by removing the root mass from the pot and placing 0.38 g millet inoculum in the bottom of the pot prior to replacing the root mass. Inoculated plants were subsequently grown at elevated temperature, 30 ± 3°C, and monitored daily for plant death due to basal stalk rot for 28 days as described previously (Underwood et al., 2021). The experimental design for each experiment was a randomized complete block design consisting of 12 blocks in which each isolate-inbred line combination was replicated 12 times and the entire experiment was repeated three times.

### *In vitro* growth of *Sclerotinia sclerotiorum* isolates

*In vitro* growth rates for 23 selected *S. sclerotiorum* isolates on PDA medium were determined at 22°C and 30°C. For each isolate, a starter culture was initiated by plating a single sclerotia on PDA and incubating at 22°C for 4 days. Plugs of 5 mm in diameter were cut from the growing edge of the colony and a single plug was transferred to each of 6 new PDA plates for each isolate. Three plates per isolate were incubated at 22°C and the remaining three plates were incubated at 30°C. After 48 h of incubation, colony diameters were measured for each plate using digital calipers and mean colony diameters across the three replicated plates from each incubation temperature were used to calculate the percent growth inhibition observed at the elevated temperature of 30°C for each isolate.

### *Sclerotinia sclerotiorum* isolate genotyping

Mycelial tissue for 227 *S. sclerotiorum* isolates was collected by growing isolates on PDA plates overlaid with a polyethersulfone filter disc (Millipore Sigma, Burlington, MA, United States) and subsequently scraping mycelium from the filter. Mycelial samples were freeze dried and 20 mg freeze dried mycelial tissue was ground to powder using a Mixer Mill MM400 (Retsch, Haan, Germany) with 2.8 mm steel grinding beads (OPS Diagnostics, Lebanon, NJ, United States). Genomic DNA was isolated from ground mycelial tissue using a Genejet Plant DNA isolation kit (Thermo Scientific, Waltham, MA, United States) according to the manufacturer’s protocol. DNA yields were quantified using a Qubit 3.0 fluorometer (Thermo Scientific, Waltham, MA, United States) and integrity was assessed by electrophoresis on 1% agarose gel. Genotyping by sequencing (GBS) was carried out by LGC Genomics (Hoddesdon, United Kingdom). GBS reads were aligned to the *S. sclerotiorum* isolate 1980 reference genome using the BWA-MEM algorithm of the Burrows-Wheeler Aligner and variants were called using the Genome Analysis Toolkit yielding an initial dataset containing 132,838 variants across 227 *S. sclerotiorum* isolates (Li and Durbin, 2009; McKenna et al., 2010; Derbyshire et al., 2017). The dataset was filtered using bcftools to remove isolates with high levels of missing data and retain variants with <30% missing data and a minimum minor allele frequency of 5% (Danecek et al., 2021). A filtered dataset of 1937 SNP variants across 219 *S. sclerotiorum* isolates was obtained for association analysis. This dataset was further filtered for phylogenetic analyses to retain only variants with <10% missing data resulting in a dataset containing 1,325 SNPs across 219 *S. sclerotiorum* isolates.

### Statistical analyses

Stem lesion lengths on HA 207 and HA 441 from α-lattice experiments were analyzed using a linear mixed model implemented in SAS version 9.4 with replicate and block (nested within replicate) considered as random effects. Significant differences among least square means for isolate lesion lengths were determined using Tukey’s post-hoc test at *p* < 0.05 (Tukey, 1949). Pearson’s correlation for isolate lesion lengths on the two inbred lines was assessed using SAS PROC CORR. Time to plant death data for basal stalk rot disease evaluations were analyzed using a generalized linear mixed model implemented in SAS PROC GLIMMIX with block and replicate considered as random effects and Tukey’s post-hoc test was used to separate means at *p* < 0.05. Data for percent growth inhibition of 23 selected isolates at 30°C were analyzed using a generalized linear mixed model with beta distribution and means were separated using Tukey’s post-hoc test at *p* < 0.05.

### Population analyses

To examine the genetic relationships among *S. sclerotiorum* isolates, a maximum likelihood phylogenetic tree was constructed with the software package RAxML version 8.0 using the GTRGAMMA model with ascertainment bias correction (ASC_GTRGAMMA) and 1,000 bootstrap analyses to obtain branch support (Stamatakis, 2014). The Interactive Tree of Life tool, iTOL,^1^ was used to visualize and annotate the maximum likelihood tree with information about host and location of collection as well as heatmaps of stem lesion aggressiveness on sunflower inbred lines HA 207 and HA 441. To assess the structure of *S. sclerotiorum* populations collected within the U.S., we used a model-free approach, discriminant analysis of principal components (DAPC), as implemented in the R package *adegenet* version 2.1.7 (Jombart, 2008; Jombart et al., 2010). The number of principal components retained for analysis was determined based on the cross-validation method. Populations were defined based on a hierarchical structure of Climate:Region:State:Host. Five regions, Northwest, Northeast, North Central, Southwest, and Southeast, were defined based on U.S. states from which the isolates were collected. Warm and cool climates were defined based on USDA plant hardiness zones, with cool climate corresponding to zones 1–6 and warm climate corresponding to zones 7 and above. This delineation defines isolates collected from areas with an average annual minimum winter temperature of above (warm) or below (cool) 0°C. Hierarchical levels with fewer than three isolates were excluded from DAPC analyses. Analysis of molecular variance (AMOVA) was performed on clone-corrected data to partition genetic variance at different levels of the hierarchy using the R package *poppr* version 2.9.3 (Kamvar et al., 2014, 2015). Statistical significance for differentiation at each hierarchical level was assessed based on 1,000 permutations. To determine the degree of genetic differentiation between isolates collected from different states or hosts, we calculated Gst (Nei, 1973) and G’st (Hedrick, 2005) using the R package *vcfR* version 1.13.0 (Knaus and Grünwald, 2017).

### Genome-wide association analyses

Least square means of stem lesion lengths observed for *S. sclerotiorum* isolates inoculated on sunflower inbred lines HA 207 and HA 441 were used with the SNP dataset containing 1937 variants to conduct a genome-wide association study for variants associated with aggressiveness using the BLINK algorithm in the R package GAPIT version 3 with population structure correction based on three principal components (Wang and Zhang, 2021). Manhattan plots and quantile-quantile plots were generated using the R package *qqman* version 0.1.8 (Turner, 2018). Genome-wide significance thresholds for association were determined using both the Bonferroni correction and the SimpleM method (Gao et al., 2008). Candidate genes were determined based on the *S. sclerotiorum* isolate 1980 reference assembly (Derbyshire et al., 2017). Candidate genes within 20 kb of an associated marker were examined using homology searches with BLASTx against the NCBI non-redundant database to determine putative functional annotations.

### Secretome and effector prediction

To determine if candidate genes associated with *S. sclerotiorum* aggressiveness are putative secreted effector proteins, predicted protein sequences from the *S. sclerotiorum* isolate 1980 reference genome assembly were analyzed using Secretool to define the predicted secretome (Cortázar et al., 2014). *S. sclerotiorum* proteins predicted to be secreted were subsequently evaluated using EffectorP version 3.0 to predict candidate apoplastic and cytoplasmic effector proteins (Sperschneider and Dodds, 2022).

## Results

### Diversity in aggressiveness of *Sclerotinia sclerotiorum* isolates on stem tissue of two sunflower inbred lines

**Figure 1:**
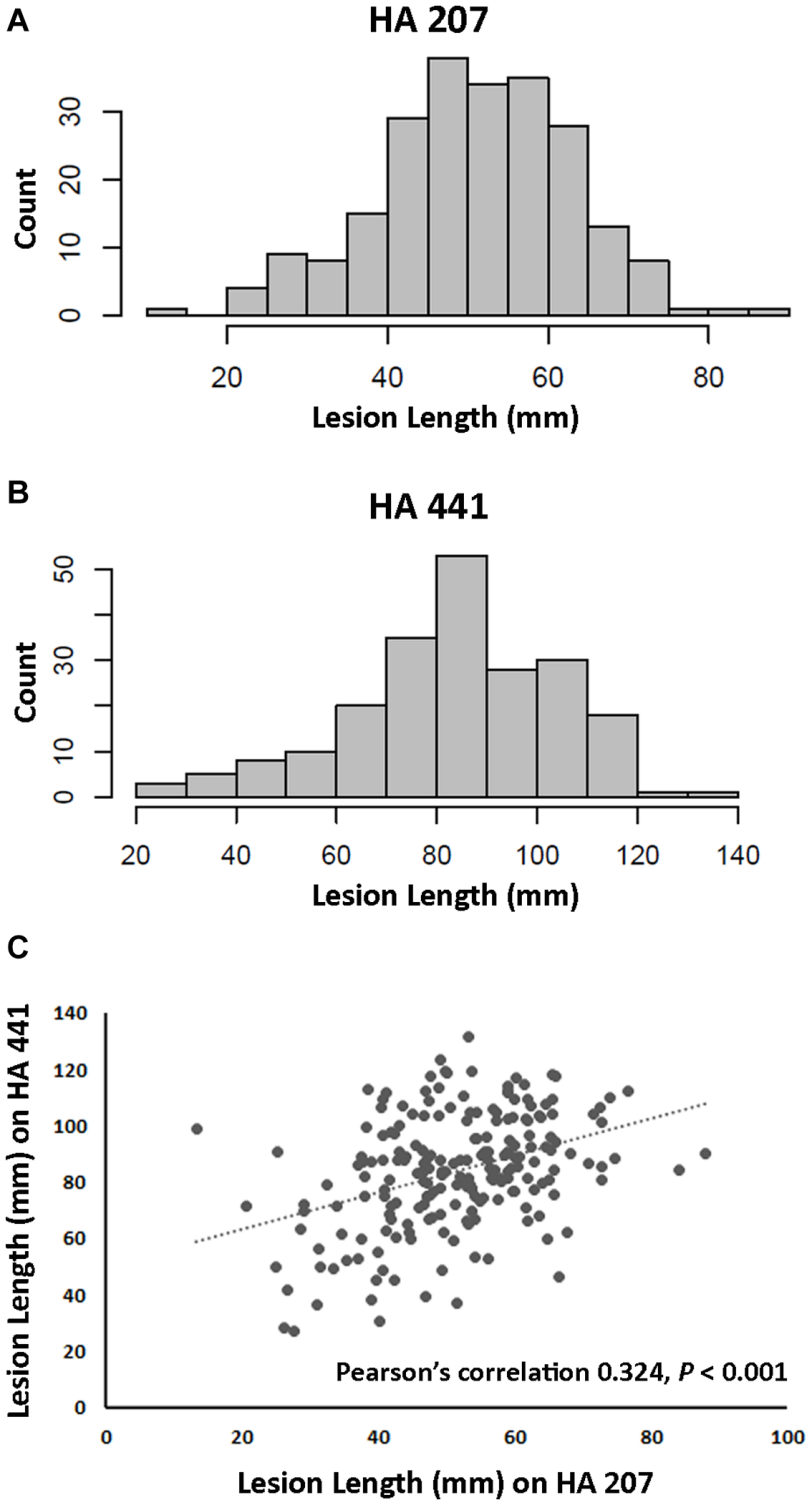
Aggressiveness of *Sclerotinia sclerotiorum* isolates on two sunflower inbred lines. **(A)** Distribution of lesion lengths for 217 isolates inoculated on inbred line HA 207. **(B)** Distribution of lesion lengths for 207 isolates inoculated on inbred line HA 441. **(C)** Scatter plot of correlation for isolate lesion lengths on HA 207 and HA 441.

A total of 219 isolates of *S. sclerotiorum* collected from diverse locations primarily within the U.S. and from 25 different susceptible host plant species were evaluated for aggressiveness on two sunflower inbred lines. Aggressiveness was determined by measuring lesion lengths at 8 dpi after inoculation on stems. Inbred lines HA 441 and HA 207 exhibit moderate and high levels, respectively, of quantitative resistance to basal stalk rot, a root-initiated disease caused by *S. sclerotiorum* (Underwood et al., 2021). However, the response of these inbred lines to stem inoculation was not known prior to this study. Significant differences (*p* < 0.001) in aggressiveness of isolates were observed on both inbred lines. and continuous distributions of lesion lengths were observed for both lines (Figures 1A,B). Mean stem lesion lengths on inbred line HA 207 ranged from approximately 13 mm to over 80 mm with an overall mean for all isolates of 51.02 mm (Figures 1A,B and Supplementary Table S1). Inbred line HA 441 appeared more susceptible to stem lesion formation, with an overall mean for all isolates of 83.55 mm and mean lesion lengths for individual isolates ranging from 20 mm to 132 mm. Mean lesion lengths for isolates on the two inbred lines were moderately and significantly (*p* < 0.001) correlated, with Pearson’s correlation of 0.324 (Figure 1C). These results indicate considerable diversity among the *S. sclerotiorum* isolates for aggressiveness in causing stem lesions on sunflower.

### Population differentiation assessed using single nucleotide polymorphism markers

**Figure 2:**
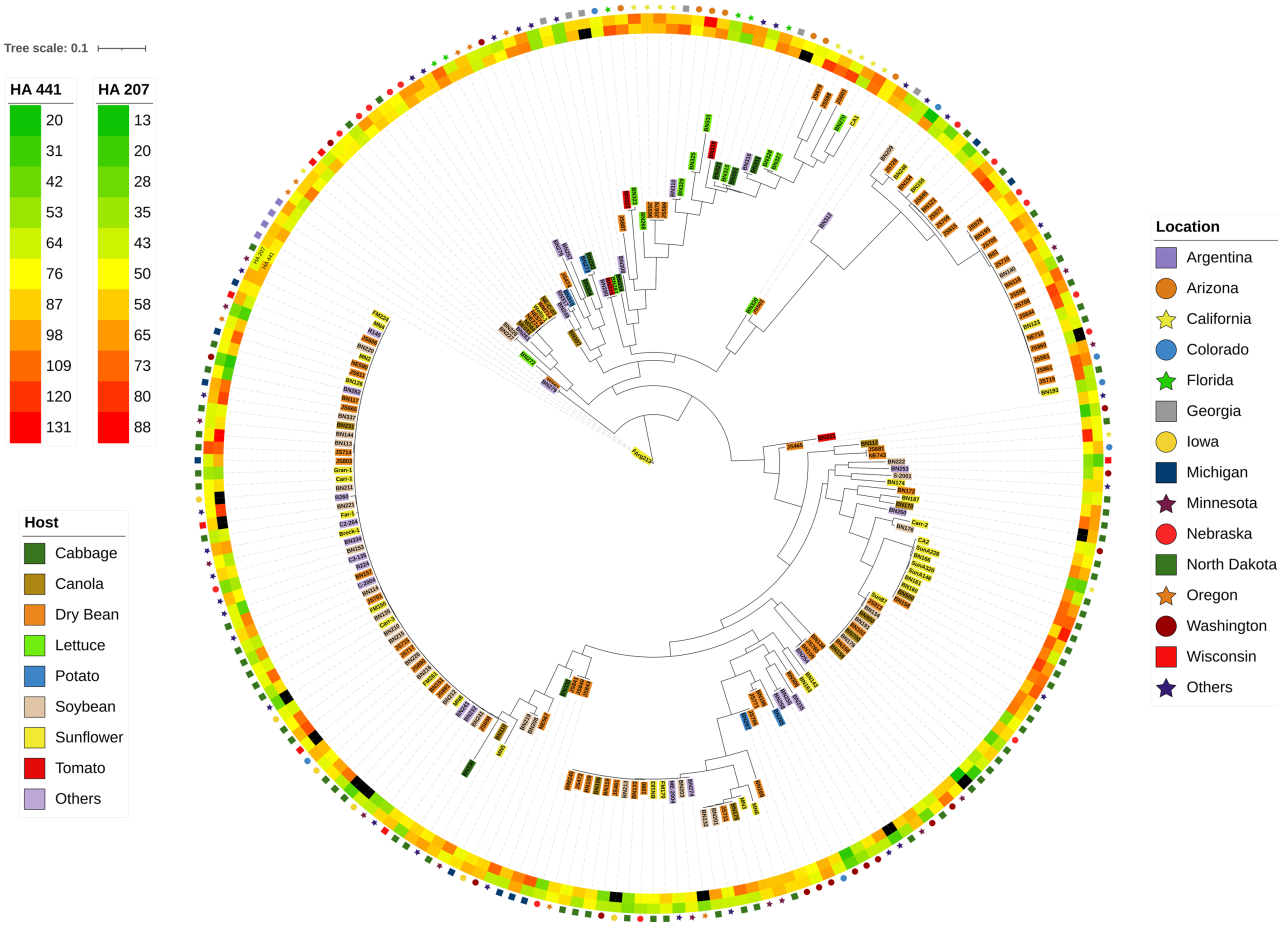
Maximum-likelihood phylogenetic tree for 219 *S. sclerotiorum* isolates inferred by RAxML using 1,325 SNP markers. The outer ring indicates the location from which isolates were collected. The second and third rings represent heatmaps for aggressiveness of isolates on sunflower inbred lines HA 207 and HA 441, respectively. Color-coded shading of isolate names indicates the host plant from which the isolate was collected.

**Figure 3:**
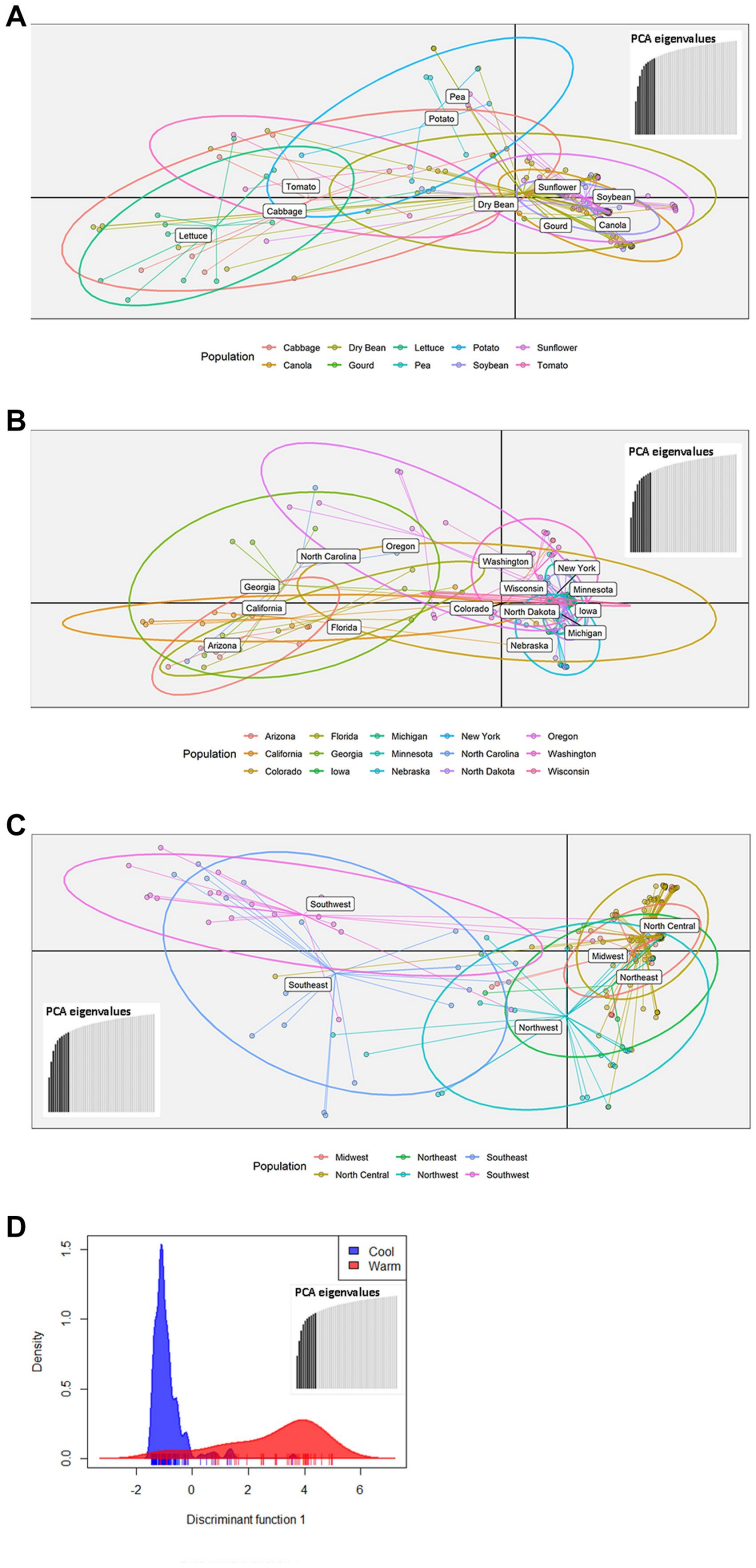
Scatterplots of discriminant analysis of principal components (DAPC) for *S. sclerotiorum* populations assigned by collection host plant **(A)**, state **(B)**, region **(C)**, or climate **(D)**. In panels **(A–C)**, DAPC was performed using 10 principal components and two discriminant functions. In panel **(D)**, DAPC was performed using 10 principal components and a single discriminant function. In panels **(A–C)**, dots represent individual isolates and ellipses indicate the area spanned by 66% of the data for a given population. Climates are defined by USDA plant hardiness zones with cool climate corresponding to zones 1–6 and warm climate corresponding to zones 7 and above.

The 219 *S. sclerotiorum* isolates were genotyped using GBS and 1,325 SNP markers were used to construct a maximum likelihood phylogenetic tree for initial evaluation of genetic relationships among isolates (Figure 2). The phylogenetic tree indicated that isolates were not grouped by host or U.S. state of collection. Four isolates collected from sunflower in Argentina clustered together and were distinct from U.S. isolates. Isolates comprising a large clonal lineage encompassing 53 of the 219 isolates were collected over a wide geographic region including 13 U.S. states across the Northwest, Northeast, Midwest, and North Central regions and collected from 9 different host plant species (Figure 2). To further investigate potential genetic differentiation among *S. sclerotiorum* populations, isolates were assigned to populations based on a hierarchy of Climate:Region:State:Host. Regions were defined as Northwest, Northeast, Midwest, North Central, Southeast, and Southwest based on geographic locations of U.S. states. Climates were defined as cool and warm based on USDA plant hardiness zones (see Materials and methods for details). We subsequently performed discriminant analysis of principal components (DAPC) for each level of the hierarchy. Grouping isolates by host of collection did not reveal clear differentiation based on host, though isolates collected from vegetable plants such as lettuce, cabbage, and tomato appeared somewhat distinct from isolates collected from field crops such as sunflower and soybean (Figure 3A). Similarly, isolate groupings by U.S. state of collection were not clearly differentiated. However, isolates collected from southern and coastal states appeared partially distinct from isolates collected from more northern and central states (Figure 3B). This trend was more apparent when isolates were grouped by region, with isolates collected from the Southwest and Southeast regions of the U.S. clustering apart, though not completely distinctly, from the northern and central regions (Figure 3C). The observation that isolates from warmer regions appeared to exhibit some degree of genetic differentiation from those collected from cooler regions prompted us to group isolates into warm and cool groupings based on USDA plant hardiness zones, as cool climate comprised of zones with average annual minimum winter temperatures below 0°F and warm climate comprised of zones with average annual minimum winter temperatures above 0°F. DAPC analysis of grouping by climate suggested some degree of differentiation based on collection in warm or cool areas (Figure 3D). AMOVA was subsequently conducted to assess population differentiation at different levels of the hierarchical assignments. Significant differentiation (*p* < 0.001) was observed between populations at the climate level, accounting for 19.54% of the observed variation (Table 1). No significant differentiation was observed between regions within climate, between states within regions, or between hosts within state (Table 1). Finally, we evaluated pairwise genetic distances among isolates between U.S. states and hosts of collection and including isolates collected in Argentina. As anticipated, pairwise Gst estimates indicated the most differentiation between isolates collected in Argentina compared to those collected in U.S. states (Supplementary Table S2). Pairwise comparisons among isolates collected from different U.S. states reinforced results obtained by DAPC analyses, where isolates collected from states in the Southeast and Southwest of the U.S. exhibited higher Gst values when compared with states from other regions (Supplementary Table S2). The lowest genetic differentiation was observed between Michigan and Minnesota (Gst = 0.00; G’st = 0.01). In comparison, the highest levels of genetic differentiation observed in comparison between U.S. states were between Iowa and Arizona (Gst = 0.46; G’st = 0.73) and Iowa and North Carolina (Gst = 0.46; G’st = 0.71). Similarly, pairwise Gst estimates were calculated to assess the presence or absence of host-specific gene flow barriers between isolates (Aldrich-Wolfe et al., 2015). Again, consistent with DAPC analyses, isolates collected from the vegetable crops lettuce, cabbage, and tomato exhibited moderately high differentiation in comparisons with isolates collected from field crops such as soybean, canola, and sunflower (Supplementary Table S3). The most closely related populations were observed between sunflower and soybean (Gst = 0.01; G’st = 0.02), and the most strongly differentiated populations were observed between soybean and lettuce (Gst = 0.29; G’st = 0.55).

**Table 1:** Analysis of molecular variance (AMOVA) comparing *Sclerotinia sclerotiorum* populations within a hierarchy of Climate/Region/State/Host using clone-corrected data.

AMOVA^a^	df^b^	SS^b^	Variation (%)	*φ* statistic	*p*
Between Climate	1	2745.99	19.54	0.195	0.001
Between Region within Climate	5	1381.69	2.67	0.033	0.116
Between State within Region	20	3433.18	2.89	0.037	0.307
Between Host within State	29	4260.41	−2.82	−0.038	0.409
Within Host	135	12901.09	77.72	0.223	0.001

a Climate is defined as warm or cool based on USDA plant hardiness zones with cool climate defined as zones 1–6 and warm climate as zones 7 and above.

b df, degrees of freedom; SS, sum of squares.

### Aggressiveness of populations assigned by U.S. state and host of collection

**Figure 4:**
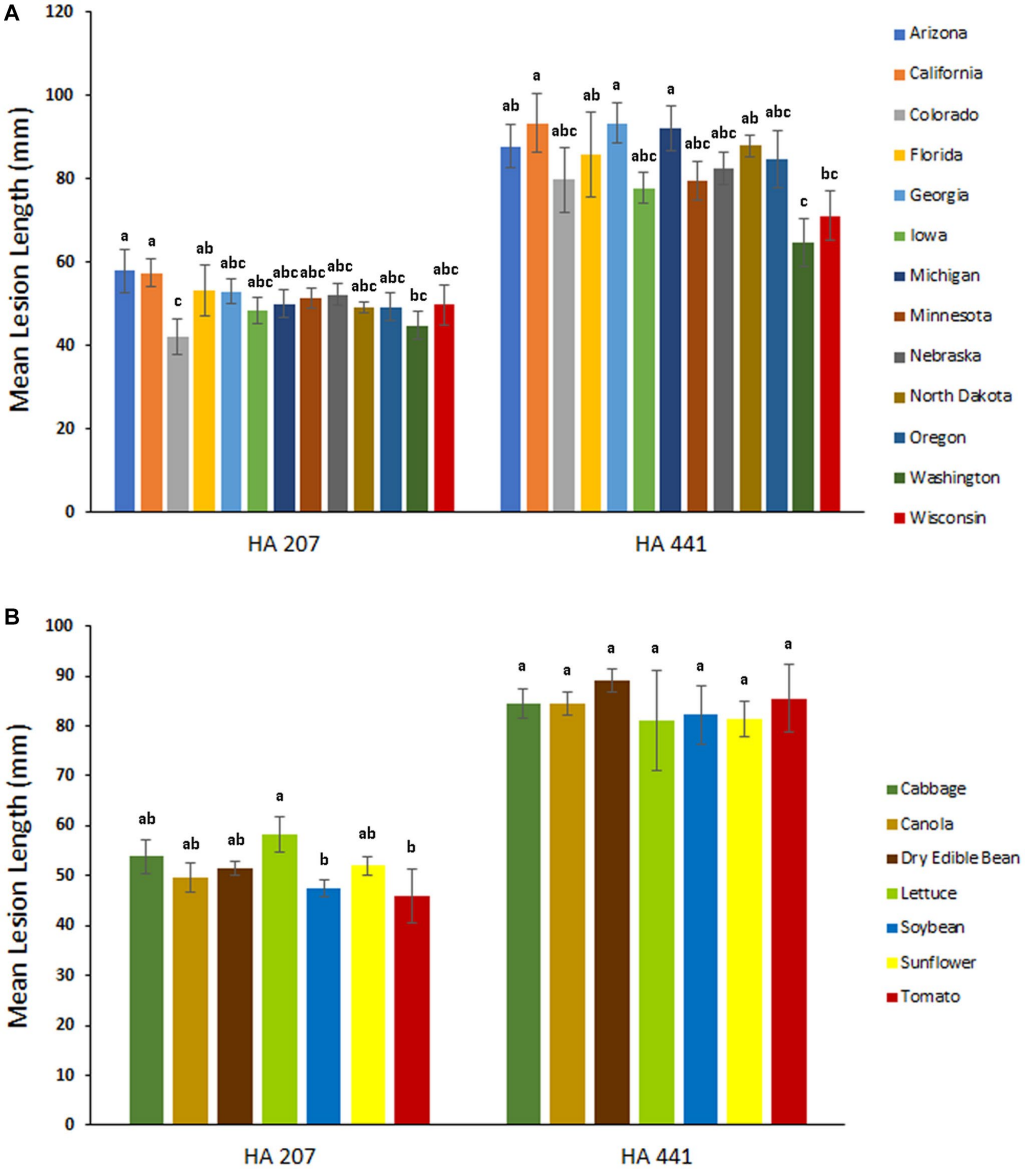
Aggressiveness of *S. sclerotiorum* isolates collected from different states **(A)** and hosts **(B)** on stems of sunflower inbred lines HA 207 and HA 441. Stem lesion lengths were measured at 8 days post-inoculation. Means indicated by the same letter are not significantly different according to analysis with a linear mixed model and Tukey’s post-hoc test (*p* < 0.05). Error bars indicate SEM.

To evaluate potential differences in aggressiveness among isolates collected from different U.S. states, we compared mean lesion lengths on stem tissues of sunflower inbred lines HA 207 and HA 441 for isolates grouped by state of collection. Significant differences in aggressiveness were observed among isolates from different states (Figure 4A). On HA 207, isolates from Arizona and California were the most aggressive in causing stem lesions while isolates from Colorado were the least aggressive. On HA 441, isolates from California and Georgia were the most aggressive and isolates from Washington were the least aggressive. Additionally, we conducted a similar analysis grouping isolates by host of collection. In general, differences in aggressiveness were less pronounced when isolates were grouped by host (Figure 4B). Significant differences were observed for mean lesion lengths on HA 207, with isolates collected from lettuce exhibiting the largest lesion lengths and isolates from soybean and tomato causing smaller lesions. In contrast, no significant differences in mean lesion lengths for isolates grouped by host of collection were observed on HA 441 (Figure 4B).

### Effects of elevated temperature on *in vitro* growth and aggressiveness in causing sunflower basal stalk rot

**Figure 5:**
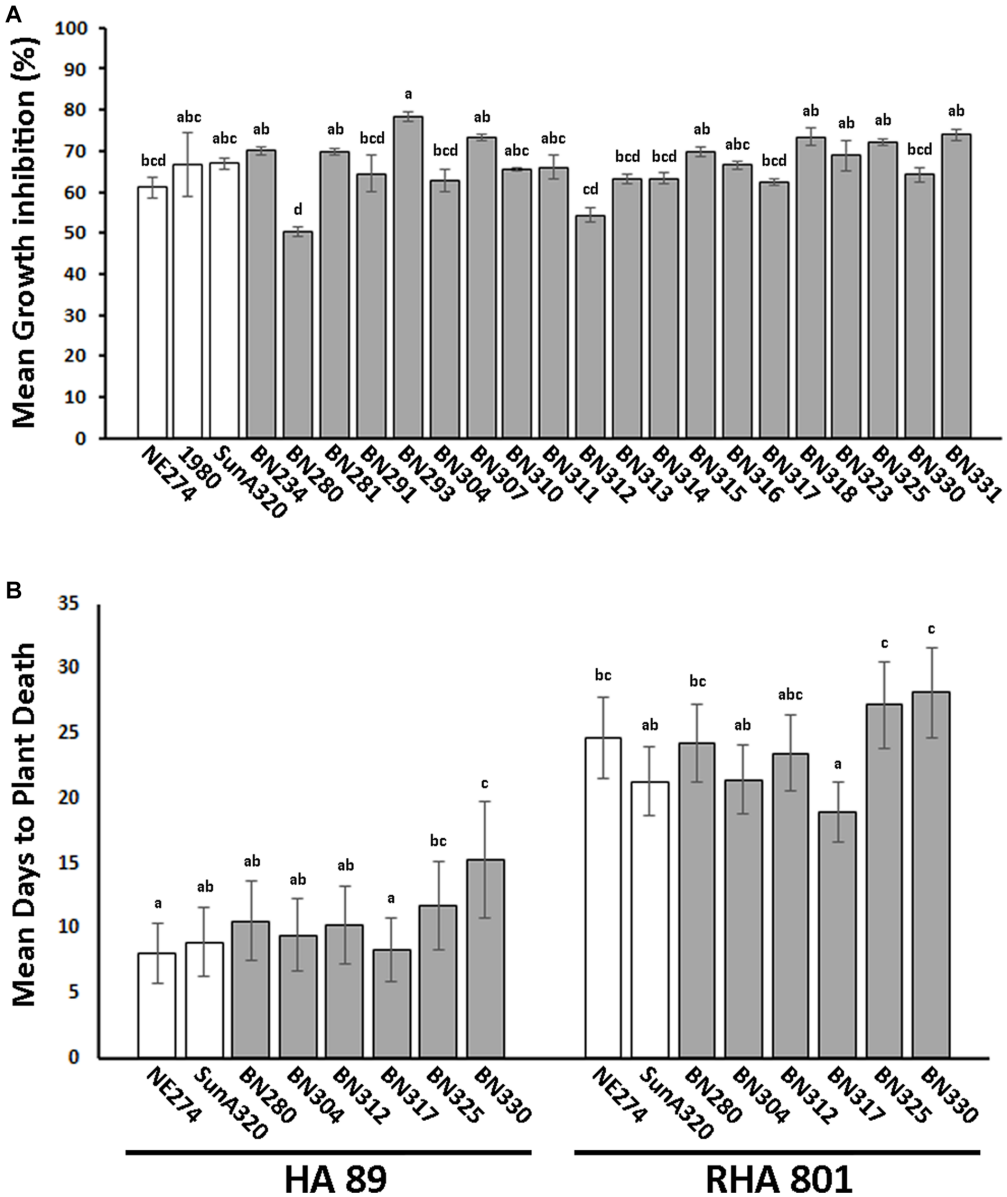
Effects of elevated temperature on *in vitro* growth and *in planta* aggressiveness of selected *S. sclerotiorum* isolates. **(A)** *In vitro* growth inhibition of *S. sclerotiorum* isolates collected from cool (white bars) or warm (gray bars) climates when grown on potato dextrose agar at 30°C compared to 22°C. Growth inhibition is expressed as % inhibition of mycelial expansion at 30°C compared to growth at 22°C. Means indicated by the same letter are not significantly different according to analysis with a linear mixed model and Tukey’s *post-hoc* test (*p* < 0.05). **(B)** Aggressiveness of isolates collected from cool (white bars) or warm (gray bars) climates in causing basal stalk rot disease on susceptible sunflower inbred line HA 89 (left) or partially resistant line RHA 801 (right) at 30°C. Aggressiveness is indicated as mean days to plant death from basal stalk rot. Means indicated by the same letter are not significantly different according to analysis with a generalized linear mixed model and Tukey’s post-hoc test (*p* < 0.05). Note that lower values for mean days to plant death indicated higher isolate aggressiveness in causing basal stalk rot. In both panels, error bars indicate SEM.

Overall, our assessments of population differentiation suggested differentiation between isolates collected in states or regions with warmer climate conditions compared to those collected from areas with cooler climates. We speculated that isolates collected from warmer climates may have increased heat tolerance, resulting in improved *in vitro* growth at elevated temperatures or increased ability to cause disease at higher temperatures. To evaluate these possibilities, we compared *in vitro* growth of 20 isolates collected from areas falling within USDA plant hardiness zones 7 and above to growth of three isolates collected from areas falling within USDA plant hardiness zones 1–6. Additionally, we compared aggressiveness in causing basal stalk rot disease on two sunflower inbred lines for six warm climate isolates and two cool climate isolates. Effects of elevated temperature on *in vitro* growth were assessed by growing replicated PDA plates for each isolate at either the typical growth temperature of 22°C or the elevated temperature of 30°C and determining the degree of inhibition of radial colony expansion caused by the higher temperature for each isolate. Significant differences in mean growth inhibition at 30°C were observed among the isolates evaluated (Figure 5A). However, we did not observe any evident trend toward improved *in vitro* growth of warm climate isolates compared to cool climate isolates. Both isolate BN280, exhibiting the least growth inhibition at 30°C, and isolate BN293, exhibiting the most growth inhibition at 30°C were collected from warm climate states (South Carolina and North Carolina, respectively; Figure 5A and Supplementary Table S1). All *in vitro* growth experiments were repeated three times with similar results. Comparison of isolate aggressiveness in causing basal stalk rot on the moderately resistant sunflower inbred line RHA 801 or the moderately susceptible line HA 89 at the elevated temperature of 30°C also revealed significant differences among isolates, but no evident trend toward higher aggressiveness of warm climate isolates at the elevated temperature (Figure 5B). None of the warm climate isolates were significantly more aggressive in causing basal stalk rot than both cool climate isolates on either tested sunflower inbred line. Overall, we found no clear evidence for improved ability of warm climate isolates to grow or cause disease at elevated temperature.

### Genome-wide association study of aggressiveness on sunflower stem tissue

**Figure 6:**
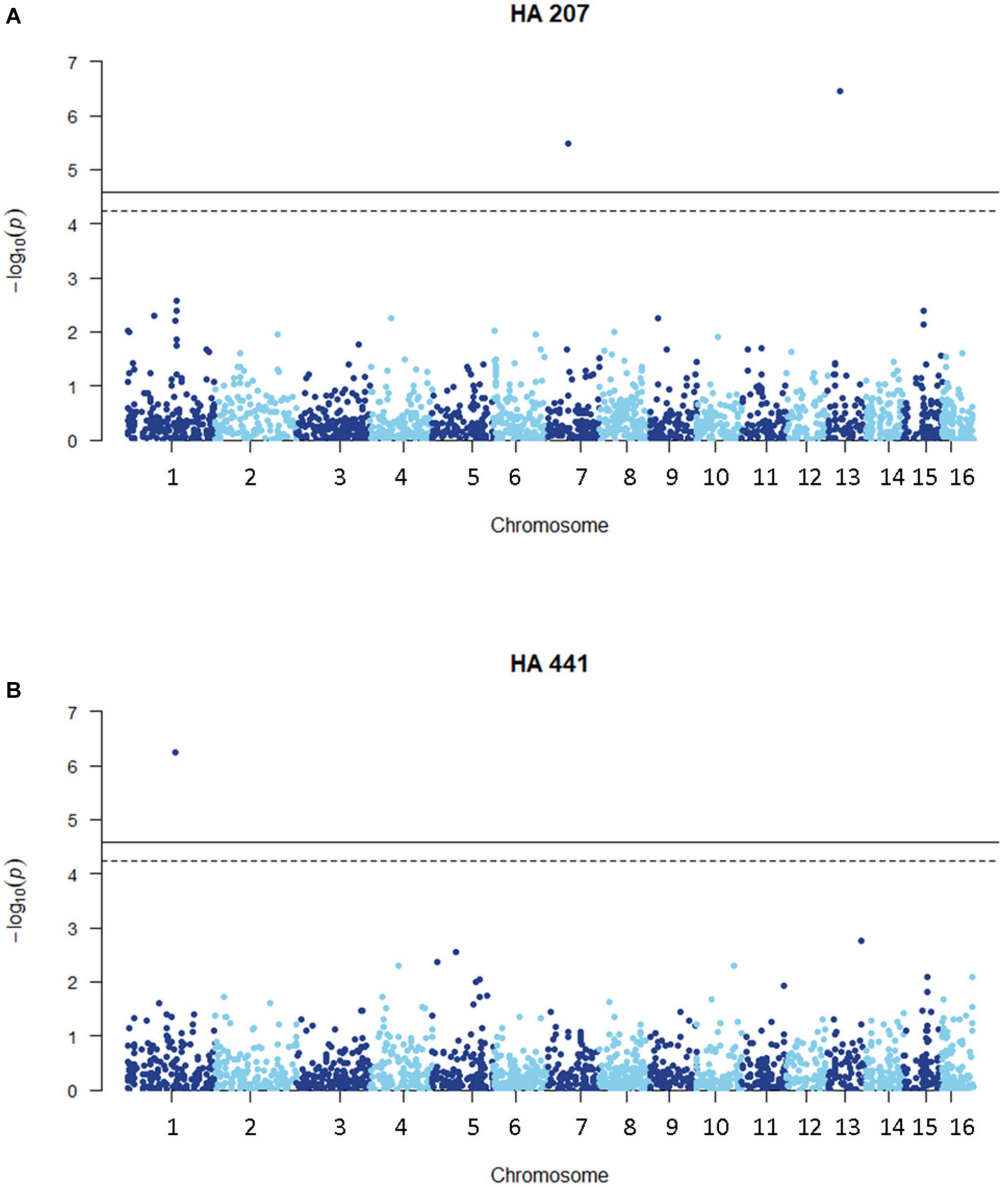
Manhattan plots of genome-wide association for aggressiveness of 219 *S. sclerotiorum* isolates in causing stem lesions on sunflower inbred lines HA 207 **(A)** or HA 441 **(B)** using 1937 SNP markers. Solid lines indicate genome-wide significance thresholds with Bonferroni multiple comparison correction and dashed lines indicate genome-wide significance thresholds determined using the SimpleM method.

We carried out a genome-wide association study (GWAS) to identify loci contributing to *S. sclerotiorum* aggressiveness using a marker set of 1937 SNPs and mean stem lesion length data for isolates inoculated on sunflower inbred lines HA 207 and HA 441. For aggressiveness on HA 207, two significant associations were identified, one each on chromosomes 7 and 13 (Figure 6A; Supplementary Figure S1A). Using aggressiveness data on HA 441, a single significant association was identified on chromosome 1 (Figure 6B; Supplementary Figure S1B). To identify potential candidate genes associated with aggressiveness, we performed homology searches for predicted genes within a 20 kb window around each associated marker based on the isolate 1980 reference genome assembly. A total of eight candidate genes were identified for the association with aggressiveness on HA 441 found on chromosome 1, including a putative cytochrome P450 with homology to pisatin demethylase and a putative MFS sugar transporter (Table 2). Eight candidate genes were identified for the association with aggressiveness on HA 207 found on chromosome 7, including a predicted kinase, a putative transcription factor, and two genes predicted to encode oligopeptide transporters (Table 2). Finally, six candidate genes were identified for the association with aggressiveness on HA 207 found on chromosome 13, including a predicted galactokinase and two genes encoding putative glycosyltransferases (Table 2). To determine if any of the identified candidate genes prospectively contributing to aggressiveness are potential effectors proteins, we used EffectorP version 3.0 (Sperschneider and Dodds, 2022) to predict candidate apoplastic and cytoplasmic effectors based on the predicted secretome. A single candidate gene on chromosome 13 with homology to glycosyltranferases encodes a protein that was predicted to be secreted and identified by EffectorP as a potential cytoplasmic effector (Table 3).

**Table 2:** Candidate genes at loci associated with *S. sclerotiorum* isolate aggressiveness on sunflower inbred lines HA 207 and HA 441 discovered by genome-wide association mapping.

MTA^a^	Chr	Position	Candidate gene	Gene position	Annotation/BLAST similarity
HA207-1	7	964805	Sscle_07g057960	955199–958306	Putative telomerase-binding Est1a protein
			Sscle_07g057970	963768–964615	Putative OPT family small oligopeptide transporter
			Sscle_07g057980	965075–966074	Putative OPT family small oligopeptide transporter
			Sscle_07g057990	967162–967870	Putative 3′–5′ exoribonuclease CSL4 protein
			Sscle_07g058000	968386–970207	Putative dihydroxyacetone kinase protein
			Sscle_07g058010	970815–971440	Putative small nuclear ribonucleoprotein SmD2
			Sscle_07g058020	971765–973337	Putative 3-hydroxyisobutyryl-coA hydrolase protein
			Sscle_07g058030	974315–978027	Putative GATA transcription factor protein
HA207-2	13	604521	Sscle_13g093530	594900–596383	Putative Ran1-like protein kinase
			Sscle_13g093540	605025–606609	Putative ring finger domain protein
			Sscle_13g093550	607876–608229	Putative glycosyltransferase family 31 protein
			Sscle_13g093560	608338–609474	Putative glycosyltransferase family 31 protein
			Sscle_13g093570	611110–612678	Putative galactokinase protein
			Sscle_13g093580	612934–614911	Putative tetratricopeptide repeat protein
HA441-1	1	2156689	Sscle_01g006240	2145377–2148208	Putative Pumilio-family RNA binding repeat protein
			Sscle_01g006250	2149150–2151948	Putative cell cycle checkpoint Rad17 protein
			Sscle_01g006260	2154154–2156729	MFS sugar transporter-like protein
			Sscle_01g006270	2157678–2159093	No similarity to known proteins
			Sscle_01g006280	2159167–2162862	Putative endonuclease/reverse transcriptase
			Sscle_01g006290	2163749–2165287	Cytochrome P450, similarity to pisatin demethylase
			Sscle_01g006300	2165739–2165909	Uncharacterized protein
			Sscle_01g006310	2166653–2167161	Putative small subunit of phenylpropionate dioxygenase

a MTA, marker-trait association.

**Table 3:** Secretion and effector predictions for candidate genes at loci associated with *S. sclerotiorum* isolate aggressiveness on sunflower inbred lines HA 207 and HA 441.

MTA^a^	Candidate gene	Annotation/BLAST similarity	Predicted secreted^b^	Predicted effector^c^
HA207-1	Sscle_07g057960	Putative telomerase-binding Est1a protein	N	N
	Sscle_07g057970	Putative OPT family small oligopeptide transporter	N	N
	Sscle_07g057980	Putative OPT family small oligopeptide transporter	N	N
	Sscle_07g057990	Putative 3′–5′ exoribonuclease CSL4 protein	N	N
	Sscle_07g058000	Putative dihydroxyacetone kinase protein	N	N
	Sscle_07g058010	Putative small nuclear ribonucleoprotein SmD2	N	N
	Sscle_07g058020	Putative 3-hydroxyisobutyryl-coA hydrolase protein	N	N
	Sscle_07g058030	Putative GATA transcription factor protein	N	N
HA207-2	Sscle_13g093530	Putative Ran1-like protein kinase	N	N
	Sscle_13g093540	Putative ring finger domain protein	N	N
	**Sscle_13g093550**	**Putative glycosyltransferase family 31 protein**	**Y**	**Y**
	Sscle_13g093560	Putative glycosyltransferase family 31 protein	N	N
	Sscle_13g093570	Putative galactokinase protein	N	N
	Sscle_13g093580	Putative tetratricopeptide repeat protein	N	N
HA441-1	Sscle_01g006240	Putative Pumilio-family RNA binding repeat protein	N	N
	Sscle_01g006250	Putative cell cycle checkpoint Rad17 protein	N	N
	Sscle_01g006260	MFS sugar transporter-like protein	N	N
	Sscle_01g006270	No similarity to known proteins	N	N
	Sscle_01g006280	Putative endonuclease/reverse transcriptase	N	N
	Sscle_01g006290	Cytochrome P450, similarity to pisatin demethylase	N	N
	Sscle_01g006300	Uncharacterized protein	N	N
	Sscle_01g006310	Putative small subunit of phenylpropionate dioxygenase	N	N

A single candidate gene predicted to be a secreted effector protein is highlighted in bold.

a MTA, marker-trait association.

b Secretion was predicted using Secretool (Cortázar et al., 2014).

c Effectors were predicted using EffectorP version 3.0 (Sperschneider and Dodds, 2022).

## Discussion

*S. sclerotiorum* causes disease and economic loss on many crop plants and is particularly damaging to agriculture in the North Central U.S., where conditions are often favorable for disease development. This pathogen causes several important diseases on cultivated sunflower that are among the most economically impactful diseases affecting U.S. sunflower production (Markell et al., 2015; Harveson et al., 2016). Resistance to *S. sclerotiorum* is quantitative and controlled by many genes in sunflower and other crop hosts. Identifying aggressive isolates that are representative of the pathogen genetic diversity and understanding population structure are important for breeding efforts to improve sunflower resistance to diseases caused by *S. sclerotiorum*. Additionally, an in depth understanding of the mechanisms through which the pathogen causes disease can aide efforts to improve host resistance and potentially lead to novel disease control strategies. Thus, the goals of this study were to utilize a diverse collection of *S. sclerotiorum* isolates spanning 27 states throughout the U.S. and 25 different host plants to characterize isolate aggressiveness in causing stem lesions on two sunflower inbred lines, investigate population characteristics using SNP markers derived from GBS, and conduct GWAS to identify loci associated with isolate aggressiveness.

Assessment of isolate lesion formation on sunflower stems revealed a broad range of aggressiveness among the studied isolates and identified isolates that were highly aggressive on one or both sunflower inbred lines used for evaluations. Variation in *S. sclerotiorum* isolate aggressiveness has been reported previously on numerous crop hosts, including sunflower (Ekins et al., 2007; Otto-Hanson et al., 2011; Taylor et al., 2015; Denton-Giles et al., 2018; Yu et al., 2020; Rather et al., 2022). We observed only a modest correlation between isolate aggressiveness on the two sunflower inbred lines, consistent with observations of genotype-isolate interactions for *S. sclerotiorum* isolates evaluated on crop hosts such as soybean, canola, and sunflower (Davar et al., 2011; Ge et al., 2012; Willbur et al., 2017; Buchwaldt et al., 2022). Resistance to *S. sclerotiorum* is quantitative rather than governed by single, dominant resistance genes characteristic of gene-for-gene interactions commonly observed in plant interactions with biotrophic pathogens. Consequently, *S. sclerotiorum* populations are not generally regarded to exhibit race structure, though one prior study reported delineation of pathotypes based on differential aggressiveness of Australian *S. sclerotiorum* isolates on eight *Brassica napus* and *Brassica juncea* accessions (Ge et al., 2012). Collectively, these results highlight the need to evaluate germplasm resources and breeding materials with multiple isolates to adequately account for pathogenic diversity and provide information about isolate aggressiveness and genetic relatedness that will be useful in selecting isolates for future germplasm evaluations.

In this study, we identified isolates that were aggressive in causing stem lesions on two sunflower inbred lines. However, *S. sclerotiorum* causes several distinct diseases on sunflower, including head rot involving infection of the sunflower capitulum (floral head) and basal stalk rot involving infection of sunflower root tissues (Markell et al., 2015; Harveson et al., 2016). Sunflower resistance to *S. sclerotiorum* is likely to be tissue specific based on lack of correlation between head rot and basal stalk rot resistance (Talukder et al., 2014). Thus, it is not clear if *S. sclerotiorum* isolates that are highly aggressive in causing stem lesions will also be aggressive on head or root tissues of sunflower and additional research may be required to identify isolates suitable for resistance screening to these forms of sunflower disease.

We used GBS to genotype our isolate collection to facilitate assessment of population characteristics and genome-wide association analysis of aggressiveness. Previous studies of *S. sclerotiorum* populations using DNA markers have generally been conducted with modest numbers of simple sequence repeat (microsatellite) markers, typically ranging from 8–16 markers evaluated (Atallah et al., 2004; Attanayake et al., 2014; Aldrich-Wolfe et al., 2015; Kamvar et al., 2017; Yu et al., 2020). In this study, we sought to evaluate potential genetic differentiation among *S. sclerotiorum* isolates collected from different regions and hosts within the U.S. using a larger number of SNP markers. Our results indicated significant differentiation between isolates collected from southern and coastal regions of the U.S. with warmer climates compared to those collected from northern and central states with colder winters. In a study of 366 *S. sclerotiorum* isolates collected from dry edible bean across 11 U.S. states and Mexico and genotyped using 11 microsatellite markers, Kamvar and colleagues noted a similar pattern of differentiation, broadly grouping isolates into those collected from coastal regions, the midwestern U.S., and Mexico (Kamvar et al., 2017). Whether this genetic differentiation is a direct result of adaptations to temperature or other climatic factors or is instead an indirect consequence of adaptations related to factors such as different cropping systems and host plants or year-round availability of hosts in warmer climates remains unclear. Several previous studies have noted genetic differentiation between regions with distinct climate conditions, and, in some cases, higher genetic variation in populations sourced from tropical or sub-tropical regions compared to temperate regions (Carbone and Kohn, 2001; Attanayake et al., 2013; Lehner and Mizubuti, 2017). The continuous availability of host plants, allowing for more reproductive cycles in tropical and sub-tropical regions has been put forth as one potential explanation for these observations (Carbone and Kohn, 2001; Attanayake et al., 2013). However, a subsequent study comparing isolates from a temperate climate region in the U.S. to those of a tropical climate region in Brazil did not observe evidence of higher levels of genetic variation in the tropical population (Lehner et al., 2017). We considered the possibility that genetically differentiated isolates from warmer regions may be better adapted for growth or plant infection at higher temperatures. However, we did not find evidence to support this notion, as isolates collected from warmer regions did not show any clear trend toward faster *in vitro* growth or more severe plant infection at elevated temperature compared to isolates collected from cooler climate regions. Additional research will be required to determine if warm climate isolates possess specific adaptations for this environment, such as improved survival or germination of sclerotia at higher temperatures or other climate-related adaptations.

We utilized data for isolate aggressiveness on stem tissues of two sunflower inbred lines along with SNP marker data to conduct a genome-wide association study for discovery of loci influencing aggressiveness. Knowledge of the virulence factors employed by *S. sclerotiorum* to cause disease on its wide range of host plants is limited and the factors contributing to variation in isolate aggressiveness are not known. Oxalic acid is regarded as a major virulence factor for this pathogen and the role of oxalic acid in *S. sclerotiorum* virulence has been studied extensively [reviewed in Xu et al. (2018)]. Additionally, several studies have noted weak to moderate correlations between oxalic acid production and isolate aggressiveness (Li et al., 2008; Willbur et al., 2017; Gill et al., 2021). A relatively small number of effector proteins have been characterized up to this point [reviewed in Derbyshire et al. (2022) and Xu et al. (2018)]. In general, most of these studies have identified putative effectors using assays for necrosis-inducing activity on host plants, mutagenesis of *S. sclerotiorum*, or bioinformatic prediction of candidate effectors, often combined with identification of genes differentially regulated during infection (Bashi et al., 2010; Guyon et al., 2014; Lyu et al., 2016; Yu et al., 2016; Westrick et al., 2019; Tang et al., 2020; Fan et al., 2021; Gupta et al., 2022; Wei et al., 2022; Newman et al., 2023). No procedures for making crosses between isolates have been developed, precluding the use of bi-parental mapping approaches to identify genetic loci influencing aggressiveness. To circumvent this limitation, GWAS represents an alternative strategy for mapping loci influencing virulence or aggressiveness, and this approach has been successfully used to identify candidate virulence factors in other phytopathogenic fungi (Gao et al., 2016; Sánchez-Vallet et al., 2018; Shakouka et al., 2022). Here, we used GWAS to identify two loci associated with aggressiveness for stem lesion formation sunflower inbred line HA 207 and one locus associated with aggressiveness on inbred line HA 441. Several interesting candidate genes were identified near the associated markers. These include a predicted cytochrome P450 with similarity to pisatin demethylase from Fusarium species, an enzyme involved in detoxification of the pea phytoalexin pisatin (Maloney and VanEtten, 1994; Coleman et al., 2011; Milani et al., 2012). Interestingly, this candidate gene was previously observed to be upregulated during infection of *B. napus*, and the authors suggested a potential role in detoxification of host defensive metabolites (Allan et al., 2019). Considering the predicted functional annotation, proximity to a marker associated with aggressiveness, and prior observations of differential expression during infection, the gene encoding this predicted P450 enzyme is a strong candidate virulence factor that likely warrants additional functional characterization. We also identified a putative glycosyltransferase that was predicted to be a secreted effector protein in analysis of the *S. sclerotiorum* predicted secretome using the EffectorP machine learning tool (Cortázar et al., 2014; Sperschneider and Dodds, 2022). It should be noted that our GWAS used a modest number of SNP markers discovered using GBS. Higher density genotyping by whole-genome resequencing of the isolates may facilitate the discovery of additional loci associated with aggressiveness. Another potential explanation for identification of only a small number of significant loci associated with aggressiveness is that this trait is controlled by many genes with effect sizes too small to detect by GWAS or requiring a larger association mapping population. In future studies, it will be interesting to evaluate aggressiveness of isolate collections such as the one described here on multiple hosts and tissue types to determine if overlapping or distinct loci influence aggressiveness on different plant hosts and tissues. The degree to which individual *S. sclerotiorum* isolates or clonal lineages are better adapted to a specific host or hosts remains unclear and evaluating aggressiveness of isolates from large collections on multiple hosts should aid in addressing this question. Future functional characterization of candidate genes identified by this study using marker-replacement mutagenesis and other tools will likely improve our understanding of the strategies used by this pathogen to cause disease on a broad range of economically important host plants. Additionally, identification of *S. sclerotiorum* genes important for aggressiveness or virulence may facilitate targeting of these genes through biotechnological approaches such as host-induced gene silencing or spray-induced gene silencing to improve disease management.

## Data availability statement

The datasets presented in this study can be found in online repositories. The names of the repository/repositories and accession number(s) can be found at: https://www.ebi.ac.uk/eva/?eva-study=PRJEB63644.

## Author contributions

RP, KB, and WU performed experiments, analyzed data, and wrote the manuscript. BN, RB, and WU designed the research project and supervised the study. BN and RB edited the manuscript. All authors contributed to the article and approved the submitted version.

## Footnotes

^1^https://itol.embl.de/

## Supplementary material

Supplementary Figure S1 Quantile-quantile plots from genome-wide association analyses for aggressiveness of 219 S. sclerotiorum isolates in causing stem lesions on sunflower inbred lines HA 207 **(A)** or HA 441 **(B)** using 1937 SNP markers.

## References

[ref1] Aldrich-Wolfe L. Travers S. Nelson B. D. Jr. (2015). Genetic variation of *Sclerotinia sclerotiorum* from multiple crops in the North Central United States. PLoS One 10:e0139188. doi: 10.1371/journal.pone.0139188, PMID: 26417989 PMC4587960

[ref2] Allan J. Regmi R. Denton-Giles M. Kamphuis L. G. Derbyshire M. C. (2019). The host generalist phytopathogenic fungus *Sclerotinia sclerotiorum* differentially expresses multiple metabolic enzymes on two different plant hosts. Sci. Rep. 9:19966. doi: 10.1038/s41598-019-56396-w, PMID: 31882688 PMC6934579

[ref3] Amouzadeh M. Darvishzadeh R. Haddadi P. Abdollahi M. B. Rezaee D. Y. (2013). Genetic analysis of partial resistance to basal stem rot (*Sclerotinia sclerotiorum*) in sunflower. Genetika 45, 737–748. doi: 10.2298/GENSR1303737A

[ref4] Aoun M. Kolmer J. A. Breiland M. Richards J. Brueggeman R. S. Szabo L. J.. (2020). Genotyping-by-sequencing for the study of genetic diversity in *Puccinia triticina*. Plant Dis. 104, 752–760. doi: 10.1094/PDIS-09-19-1890-RE, PMID: 31910116

[ref5] Atallah Z. K. Larget B. Chen X. Johnson D. A. (2004). High genetic diversity, phenotypic uniformity, and evidence of outcrossing in *Sclerotinia sclerotiorum* in the Columbia Basin of Washington State. Phytopathology 94, 737–742. doi: 10.1094/PHYTO.2004.94.7.737, PMID: 18943906

[ref6] Attanayake R. N. Porter L. Johnson D. A. Chen W. (2012). Genetic and phenotypic diversity and random association of DNA markers of isolates of the fungal plant pathogen *Sclerotinia sclerotiorum* from soil on a fine geographic scale. Soil Biol. Biochem. 55, 28–36. doi: 10.1016/j.soilbio.2012.06.002

[ref1002] Attanayake R. N. Carter P. A. Jiang D. Del Río Mendoza L. del Río-Mendoza L. Chen W.. (2013). Sclerotinia sclerotiorum populations infecting canola from China and the United States are genetically and phenotypically distinct. Phytopathology. 103, 750–761. doi: 10.1094/PHYTO-07-12-0159-R, PMID: 23464902

[ref7] Attanayake R. N. Tennekoon V. Johnson D. A. Porter L. D. del Río-Mendoza L. Jiang D.. (2014). Inferring outcrossing in the homothallic fungus *Sclerotinia sclerotiorum* using linkage disequilibrium decay. Heredity 113, 353–363. doi: 10.1038/hdy.2014.37, PMID: 24781807 PMC4181068

[ref8] Bartoli C. Roux F. (2017). Genome-wide association studies in plant pathosystems: toward an ecological genomics approach. Front. Plant Sci. 8:763. doi: 10.3389/fpls.2017.00763, PMID: 28588588 PMC5441063

[ref9] Bashi Z. D. Hegedus D. D. Buchwaldt L. Rimmer R. Borhan M. H. (2010). Expression and regulation of *Sclerotinia sclerotiorum* necrosis and ethylene-inducing peptides (NEPs). Mol. Plant Pathol. 11, 43–53. doi: 10.1111/j.1364-3703.2009.00571.x 20078775 PMC6640525

[ref10] Boland G. J. Hall R. (1994). Index of plant hosts of *Sclerotinia sclerotiorum*. Can. J. Plant Pathol. 16, 93–108. doi: 10.1080/07060669409500766

[ref11] Bolton M. D. Thomma B. P. H. J. Nelson B. D. (2006). *Sclerotinia sclerotiorum* (Lib.) de Bary: biology and molecular traits of a cosmopolitan pathogen. Mol. Plant Pathol. 7, 1–16. doi: 10.1111/j.1364-3703.2005.00316.x, PMID: 20507424

[ref12] Brown J. K. M. (1995). “Pathogens’ responses to the management of disease resistance genes” in Advances in plant pathology. eds. Andrews J. H. Tommerup I. C. (London, UK: Academic Press), 75–102.

[ref13] Buchwaldt L. Garg H. Puri K. D. Durkin J. Adam J. Harrington M.. (2022). Sources of genomic diversity in the self-fertile plant pathogen, *Sclerotinia sclerotiorum*, and consequences for resistance breeding. PLoS One 17:e0262891. doi: 10.1371/journal.pone.0262891, PMID: 35130285 PMC8820597

[ref14] Carbone I. Anderson J. B. Kohn L. M. (1999). Patterns of descent in clonal lineages and their multilocus fingerprints are resolved with combined gene genealogies. Evolution 53, 11–21. doi: 10.1111/j.1558-5646.1999.tb05329.x, PMID: 28565180

[ref15] Carbone I. Kohn L. M. (2001). A microbial population-species interface: nested cladistic and coalescent inference with multilocus data. Mol. Ecol. 10, 947–964. doi: 10.1046/j.1365-294x.2001.01244.x, PMID: 11348503

[ref16] Coleman J. J. Wasmann C. C. Usumi T. White G. J. Temporini E. D. McCluskey K.. (2011). Characterization of the gene encoding pisatin demethylase (FoPDA1) in *Fusarium oxysporum*. Mol. Plant-Microbe Interact. 24, 1482–1491. doi: 10.1094/MPMI-05-11-0119, PMID: 22066900

[ref17] Cortázar A. R. Aransay A. M. Alfaro M. Oguiza J. A. Lavín J. L. (2014). SECRETOOL: integrated secretome analysis tool for fungi. Amino Acids 46, 471–473. doi: 10.1007/s00726-013-1649-z, PMID: 24370983

[ref18] Cubeta M. A. Cody B. R. Kohli Y. Kohn L. M. (1997). Clonality in *Sclerotinia sclerotiorum* on infected cabbage in eastern North Carolina. Phytopathology 87, 1000–1004. doi: 10.1094/PHYTO.1997.87.10.1000, PMID: 18945032

[ref19] Danecek P. Bonfield J. K. Liddle J. Marshall J. Ohan V. Pollard M. O.. (2021). Twelve years of SAMtools and BCFtools. Gigascience 10:giab008. doi: 10.1093/gigascience/giab008, PMID: 33590861 PMC7931819

[ref20] Davar R. Darvishzadeh R. Majd A. (2011). Genotype-isolate interactions for resistance to *Sclerotinia sclerotiorum* in sunflower. Phytopathol. Mediterr. 50, 442–449. doi: 10.14601/Phytopathol_Mediterr-9505

[ref21] Davar R. Darvishzadeh R. Majd A. Ghosta Y. Sarrafi A. (2010). QTL mapping of partial resistance to basal stem rot in sunflower using recombinant inbred lines. Phytopathol. Mediterr. 49, 330–341. doi: 10.14601/Phytopathol_Mediterr-8374

[ref22] Denton-Giles M. Derbyshire M. C. Khentry Y. Buchwaldt L. Kamphuis L. G. (2018). Partial stem resistance in *Brassica napus* to highly aggressive and genetically diverse *Sclerotinia sclerotiorum* isolates from Australia. Can. J. Plant Pathol. 40, 551–561. doi: 10.1080/07060661.2018.1516699

[ref23] Derbyshire M. Denton-Giles M. Hegedus D. Seifbarghy S. Rollins J. van Kan J.. (2017). The complete genome sequence of the phytopathogenic fungus *Sclerotinia sclerotiorum* reveals insights into the genome architecture of broad host range pathogens. Genome Biol. Evol. 9, 593–618. doi: 10.1093/gbe/evx030, PMID: 28204478 PMC5381539

[ref24] Derbyshire M. C. Newman T. E. Khentry Y. Owolabi Taiwo A. (2022). The evolutionary and molecular features of the broad-host-range plant pathogen *Sclerotinia sclerotiorum*. Mol. Plant Pathol. 23, 1075–1090. doi: 10.1111/mpp.13221, PMID: 35411696 PMC9276942

[ref25] Ekins M. G. Aitken E. A. B. Goulter K. C. (2007). Aggressiveness among isolates of *Sclerotinia sclerotiorum* from sunflower. Australas. Plant Pathol. 36, 580–586. doi: 10.1071/AP07062

[ref26] Elshire R. J. Glaubitz J. C. Sun Q. Poland J. A. Kawamoto K. Buckler E. S.. (2011). A robust, simple genotyping-by-sequencing (GBS) approach for high diversity species. PLoS One 6:e19379. doi: 10.1371/journal.pone.0019379, PMID: 21573248 PMC3087801

[ref27] Fan H. Yang W. Nie J. Zhang W. Wu J. Wu D.. (2021). A novel effector protein SsERP1 inhibits plant ethylene signaling to promote *Sclerotinia sclerotiorum* infection. J. Fungi 7:825. doi: 10.3390/jof7100825, PMID: 34682246 PMC8537369

[ref28] Filippi C. V. Zubrzycki J. E. Di Rienzo J. A. Quiroz F. J. Puebla A. F. Alvarez D.. (2020). Unveiling the genetic basis of sclerotinia head rot resistance in sunflower. BMC Plant Biol. 20:322. doi: 10.1186/s12870-020-02529-7, PMID: 32641108 PMC7346337

[ref29] Gao Y. Liu Z. Faris J. D. Richards J. Brueggeman R. S. Li X.. (2016). Validation of genome-wide association studies as a tool to identify virulence factors in *Parastagonospora nodorum*. Phytopathology 106, 1177–1185. doi: 10.1094/PHYTO-02-16-0113-FI, PMID: 27442533

[ref30] Gao X. Starmer J. Martin E. R. (2008). A multiple testing correction method for genetic association studies using correlated single nucleotide polymorphisms. Genet. Epidemiol. 32, 361–369. doi: 10.1002/gepi.20310 18271029

[ref31] Ge X. T. Li Y. P. Wan Z. J. You M. P. Finnegan P. M. Banga S. S.. (2012). Delineation of *Sclerotinia sclerotiorum* pathotypes using differential resistance responses on *Brassica napus* and *B. juncea* genotypes enables identification of resistance to prevailing pathotypes. Field Crop Res. 127, 248–258. doi: 10.1016/j.fcr.2011.11.022

[ref32] Gill R. Sandu P. S. Sharma S. Sharma P. (2021). Pathogenicity determinants of *Sclerotinia sclerotiorum* and their association to its aggressiveness on *Brassica juncea*. Plant Pathol. J. 37, 365–374. doi: 10.5423/PPJ.OA.03.2021.0036, PMID: 34365748 PMC8357566

[ref33] Gulya T. Harveson R. Mathew F. Block C. Thompson S. Kandel H.. (2019). Comprehensive disease survey of U.S. sunflower: disease trends, research priorities and unanticipated impacts. Plant Dis. 103, 601–618. doi: 10.1094/PDIS-06-18-0980-FE, PMID: 30789318

[ref34] Guo X. Stotz H. U. (2007). Defense against *Sclerotinia sclerotiorum* in Arabidopsis is dependent on jasmonic acid, salicylic acid, and ethylene signaling. Mol. Plant-Microbe Interact. 20, 1384–1395. doi: 10.1094/MPMI-20-11-1384 17977150

[ref35] Gupta N. C. Yadav S. Arora S. Mishra D. C. Dudhlakot N. Gaikwad K.. (2022). Draft genome sequencing and secretome profiling of *Sclerotinia sclerotiorum* revealed effector repertoire diversity and allied broad-host range necrotrophy. Sci. Rep. 12:21855. doi: 10.1038/s41598-022-22028-z, PMID: 36528657 PMC9759525

[ref36] Guyon K. Balagué C. Roby D. Raffaele S. (2014). Secretome analysis reveals effector candidates associated with broad host range necrotrophy in the fungal plant pathogen *Sclerotinia sclerotiorum*. BMC Genomics 15:336. doi: 10.1186/1471-2164-15-336, PMID: 24886033 PMC4039746

[ref37] Harveson R. M. Markell S. G. Block C. C. Gulya T. J. (2016) Compendium of sunflower diseases and pests. APS Press, St. Paul, MN.

[ref38] Hedrick P. W. (2005). A standardized genetic differentiation measure. Evolution 59, 1633–1638. doi: 10.1111/j.0014-3820.2005.tb01814.x, PMID: 16329237

[ref39] Heffer Link V. Johnson K. B. (2007). White mold. Plant Health Inst. doi: 10.1094/PHI-I-2007-0809-01

[ref40] Jombart T. (2008). *adegenet*: a R package for the multivariate analysis of genetic markers. Bioinformatics 24, 1403–1405. doi: 10.1093/bioinformatics/btn129, PMID: 18397895

[ref41] Jombart T. Devillard S. Balloux F. (2010). Discriminant analysis of principal components: a new method for the analysis of genetically structured populations. BMC Genet. 11:94. doi: 10.1186/1471-2156-11-94, PMID: 20950446 PMC2973851

[ref42] Kamvar Z. N. Amaradasa B. S. Jhala R. McCoy S. Steadman J. R. Everhart S. E. (2017). Population structure and phenotypic variation of *Sclerotinia sclerotiorum* from dry bean (*Phaseolus vulgaris*) in the United States. PeerJ 5:e4152. doi: 10.7717/peerj.4152, PMID: 29230376 PMC5723432

[ref43] Kamvar Z. N. Brooks J. C. Grünwald N. J. (2015). Novel R tools for analysis of genome-wide population genetic data with emphasis on clonality. Front. Genet. 6:208. doi: 10.3389/fgene.2015.00208, PMID: 26113860 PMC4462096

[ref44] Kamvar Z. N. Tabima J. F. Grünwald N. J. (2014). Poppr: an R package for genetic analysis of populations with clonal, partially clonal, and/or sexual reproduction. PeerJ 2:e281. doi: 10.7717/peerj.281, PMID: 24688859 PMC3961149

[ref45] Knaus B. J. Grünwald N. J. (2017). VCFR: a package to manipulate and visualize variant call format data in R. Mol. Ecol. Resourc. 17, 44–53. doi: 10.1111/1755-0998.12549, PMID: 27401132

[ref46] Kohli Y. Morrall R. A. A. Anderson J. B. Kohn L. M. (1992). Local and trans-Canadian clonal distribution of *Sclerotinia sclerotiorum* on canola. Phytopathology 82, 875–880. doi: 10.1094/Phyto-82-875

[ref47] Leboldus J. M. Kinzer K. Richards J. Ya Z. Yan C. Friesen T. L.. (2015). Genotype-by-sequencing of the plant-pathogenic fungi *Pyrenophora teres* and *Sphaerulina musiva* utilizing Ion Torrent sequence technology. Mol. Plant Pathol. 16, 623–632. doi: 10.1111/mpp.12214, PMID: 25346350 PMC6638358

[ref48] Lehner M. S. de Paula Júnior T. J. Del Ponte E. M. Mizubuti E. S. G. Pethybridge S. J. (2017). Independently founded populations of *Sclerotinia sclerotiorum* from a tropical and a temperate region have similar genetic structure. PLoS One 12:e0173915. doi: 10.1371/journal.pone.0173915, PMID: 28296968 PMC5352009

[ref49] Lehner M. S. Mizubuti E. S. G. (2017). Are *Sclerotinia sclerotiorum* populations from the tropics more variable than those from subtropical and temperate zones? Trop. Plant Pathol. 42, 61–69. doi: 10.1007/s40858-016-0125-1

[ref50] Li H. Durbin R. (2009). Fast and accurate short read alignment with Burrows–Wheeler transform. Bioinformatics 25, 1754–1760. doi: 10.1093/bioinformatics/btp324, PMID: 19451168 PMC2705234

[ref51] Li Z. Zhang M. Wang Y. Li R. Dilantha Fernando W. G. (2008). Mycelial compatibility group and pathogenicity variation of *Sclerotinia sclerotiorum* populations in sunflower from China, Canada, and England. Plant Pathol. J. 7, 131–139. doi: 10.3923/ppj.2008.131.139

[ref52] Lyu X. Shen C. Fu Y. Xie J. Jiang D. Li G.. (2016). A small secreted virulence-related protein is essential for the necrotrophic interactions of *Sclerotinia sclerotiorum* with its host plants. PLoS Pathog. 12:e1005435. doi: 10.1371/journal.ppat.1005435, PMID: 26828434 PMC4735494

[ref53] Maloney A. P. VanEtten H. D. (1994). A gene from the fungal plant pathogen *Nectria haematococca* that encodes the phytoalexin-detoxifying enzyme pisatin demethylase defines a new cytochrome P450 family. Mol. Gen. Genet. 243, 506–514. doi: 10.1007/BF00284198, PMID: 8208242

[ref54] Malvarez G. Carbone I. Grunwald N. J. Subbarao K. V. Schafer M. Kohn L. M. (2007). New populations of *Sclerotinia sclerotiorum* from lettuce in California and peas and lentils in Washington. Phytopathology 97, 470–483. doi: 10.1094/PHYTO-97-4-0470, PMID: 18943288

[ref55] Markell S. G. Harveson R. M. Block C. C. Gulya T. J. (2015). “Sunflower diseases” in Sunflower: chemistry, production, processing and utilization. eds. Martínez Force E. Dunford N. T. Salas J. J. (Urbana, IL: AOCS Press), 83–128.

[ref56] McDonald B. A. Linde C. (2002). Pathogen population genetics, evolutionary potential, and durable resistance. Annu. Rev. Phytopathol. 40, 349–379. doi: 10.1146/annurev.phyto.40.120501.101443 12147764

[ref57] McKenna A. Hanna M. Banks E. Sivachenko A. Cibulskis K. Kernytsky A.. (2010). The genome analysis toolkit: a MapReduce framework for analyzing next-generation DNA sequencing data. Genome Res. 20, 1297–1303. doi: 10.1101/gr.107524.110, PMID: 20644199 PMC2928508

[ref58] Milani N. A. Lawrence D. P. Arnold A. E. VanEtten H. D. (2012). Origin of pisatin demethylase (PDA) in the genus *Fusarium*. Fungal Genet. Biol. 49, 933–942. doi: 10.1016/j.fgb.2012.08.007, PMID: 22985693

[ref59] Miller J. F. Gulya T. J. (2006). Registration of two restorer (RHA 439 and RHA 440) and one maintainer (HA 441) sclerotinia tolerant oilseed sunflower germplasms. Crop Sci. 46, 482–483. doi: 10.2135/cropsci2005.04-0009

[ref60] Nei M. (1973). Analysis of gene diversity in subdivided populations. Proc. Natl. Acad. Sci. U. S. A. 70, 3321–3323. doi: 10.1073/pnas.70.12.3321, PMID: 4519626 PMC427228

[ref61] Newman T. E. Kim H. Khentry Y. Sohn K. H. Derbyshire M. C. Kamphuis L. G. (2023). The broad host range pathogen *Sclerotinia sclerotiorum* produces multiple effector proteins that induce host cell death intracellularly. Mol. Plant Pathol. 24, 866–881. doi: 10.1111/mpp.13333, PMID: 37038612 PMC10346375

[ref62] Otto-Hanson L. Steadman J. R. Higgins R. Eskridge K. M. (2011). Variation in *Sclerotinia sclerotiorum* from multisite resistance screening locations. Plant Dis. 95, 1370–1377. doi: 10.1094/PDIS-11-10-0865, PMID: 30731780

[ref63] Poland J. A. Brown P. J. Sorrells M. E. Jannink J. L. (2012). Development of high-density genetic maps for barley and wheat using a novel two-enzyme genotyping-by-sequencing approach. PLoS One 7:e32253. doi: 10.1371/journal.pone.0032253, PMID: 22389690 PMC3289635

[ref64] Qi L. Long Y. Talukder Z. I. Seiler G. J. Block C. C. Gulya T. J. (2016). Genotyping-by-sequencing uncovers the introgression alien segments associated with sclerotinia basal stalk rot resistance from wild species—I. *Helianthus argophyllus* and *H. petiolaris*. Front. Genet. 7:219. doi: 10.3389/fgene.2016.00219, PMID: 28083014 PMC5183654

[ref65] Rather R. A. Ahanger F. A. Ahanger S. A. Basu U. Wani M. A. Rashid Z.. (2022). Morpho-cultural and pathogenic variability of *Sclerotinia sclerotiorum* causing white mold of common beans in temperate climate. J. Fungi 8:755. doi: 10.3390/jof8070755, PMID: 35887510 PMC9316490

[ref66] Roath W. W. Miller J. F. Gulya T. J. (1981). Registration of RHA 801 sunflower germplasm. Crop Sci. 21:479. doi: 10.2135/cropsci1981.0011183X002100030041x

[ref67] Sánchez-Vallet A. Hartmann F. E. Marcel T. C. Croll D. (2018). Nature’s genetic screens: using genome-wide association studies for effector discovery. Mol. Plant Pathol. 19, 3–6. doi: 10.1111/mpp.12592, PMID: 29226559 PMC6638067

[ref68] Schafer M. R. Kohn L. M. (2006). An optimized method for mycelial compatibility testing in *Sclerotinia sclerotiorum*. Mycologia 98, 593–597. doi: 10.3852/mycologia.98.4.593, PMID: 17139852

[ref69] Seiler G. J. Misar C. G. Gulya T. J. Underwood W. R. Flett B. C. Gilley M. A.. (2017). Identification of novel sources of resistance to sclerotinia basal stalk rot in South African sunflower germplasm. Plant Health Prog. 18, 87–90. doi: 10.1094/PHP-01-17-0007-RS

[ref70] Shakouka M. A. Gurjar M. S. Aggerwal R. Saharan M. S. Gogoi R. Kumar N. B.. (2022). Genome-wide association mapping of virulence genes in wheat Karnal bunt fungus *Tilletia indica* using double digest restriction-site associated DNA-genotyping by sequencing approach. Front. Microbiol. 13:852727. doi: 10.3389/fmicb.2022.852727, PMID: 35633675 PMC9139842

[ref71] Sperschneider J. Dodds P. N. (2022). EffectorP 3.0: prediction of apopolastic and cytoplasmic effectors in fungi and oomycetes. Mol. Plant-Microbe Interact. 35, 146–156. doi: 10.1094/MPMI-08-21-0201-R, PMID: 34698534

[ref72] Stafford R. E. Thompson T. E. (1983). Registration of sunflower parental line HA 207. Crop Sci. 23:195. doi: 10.2135/cropsci1983.0011183X002300010091x

[ref73] Stamatakis A. (2014). RAxML version 8: a tool for phylogenetic analysis and post-analysis of large phylogenies. Bioinformatics 30, 1312–1313. doi: 10.1093/bioinformatics/btu033, PMID: 24451623 PMC3998144

[ref74] Talukder Z. I. Hulke B. S. Marek L. F. Gulya T. J. (2014). Sources of resistance to sunflower diseases in a global collection of domesticated USDA plant introductions. Crop Sci. 54, 694–705. doi: 10.2135/cropsci2013.07.0506

[ref75] Talukder Z. I. Seiler G. J. Song Q. Ma G. Qi L. (2016). SNP discovery and QTL mapping of sclerotinia basal stalk rot resistance in sunflower using genotyping-by-sequencing. Plant Genome 9:plantgenome2016.03.0035. doi: 10.3835/plantgenome2016.03.0035, PMID: 27902793

[ref76] Talukder Z. I. Underwood W. Misar C. G. Seiler G. J. Cai X. Li X.. (2022a). A quantitative genetic study of sclerotinia head rot resistance introgressed from the wild perennial *Helianthus maximiliani* into cultivated sunflower (*Helianthus annuus* L.). Int. J. Mol. Sci. 23:7727. doi: 10.3390/ijms23147727, PMID: 35887074 PMC9321925

[ref77] Talukder Z. I. Underwood W. Misar C. G. Seiler G. J. Cai X. Li X.. (2022b). Genomic insights into sclerotinia basal stalk rot resistance introgressed from wild *Helianthus praecox* into cultivated sunflower (*Helianthus annuus* L.). Front. Plant Sci. 13:840954. doi: 10.3389/fpls.2022.840954, PMID: 35665155 PMC9158519

[ref78] Talukder Z. I. Underwood W. Misar C. G. Seiler G. J. Liu Y. Li X.. (2021). Unraveling the sclerotinia basal stalk rot resistance derived from wild *Helianthus argophyllus* using a high-density single nucleotide polymorphism linkage map. Front. Plant Sci. 11:617920. doi: 10.3389/fpls.2020.617920, PMID: 33613588 PMC7886805

[ref79] Tang L. Yang G. Ma M. Liu X. Li B. Xie J.. (2020). An effector of a necrotrophic fungal pathogen targets the calcium-sensing receptor in chloroplasts to inhibit host resistance. Mol. Plant Pathol. 21, 686–701. doi: 10.1111/mpp.12922, PMID: 32105402 PMC7170781

[ref80] Taylor A. Coventry E. Jones J. E. Clarkson J. P. (2015). Resistance to a highly aggressive isolate of *Sclerotinia sclerotiorum* in a *Brassica napus* diversity set. Plant Pathol. 64, 932–940. doi: 10.1111/ppa.12327

[ref81] Tukey J. (1949). Comparing individual means in the analysis of variance. Biometrics 5, 99–114. doi: 10.2307/3001913 18151955

[ref82] Turner S. D. (2018). qqman: an R package for visualizing GWAS results using Q-Q and Manhattan plots. J. Open Source Softw. 3:731. doi: 10.21105/joss.00731

[ref83] Underwood W. Misar C. G. Block C. C. Gulya T. J. Talukder Z. Hulke B. S.. (2021). A greenhouse method to evaluate sunflower quantitative resistance to basal stalk rot caused by *Sclerotinia sclerotiorum*. Plant Dis. 105, 464–472. doi: 10.1094/PDIS-08-19-1790-RE, PMID: 33264029

[ref84] Wang J. Zhang Z. (2021). GAPIT version 3: boosting power and accuracy for genomic association and prediction. Genomics Proteomics Bioinformatics 19, 629–640. doi: 10.1016/j.gpb.2021.08.005, PMID: 34492338 PMC9121400

[ref85] Wei W. Xu L. Peng H. Zhu W. Tanaka K. Cheng J.. (2022). A fungal extracellular effector inactivates plant polygalacturonase-inhibiting protein. Nat. Commun. 13:2213. doi: 10.1038/s41467-022-29788-2, PMID: 35468894 PMC9038911

[ref86] Westrick N. M. Ranjan A. Jain S. Grau C. R. Smith D. L. Kabbage M. (2019). Gene regulation of *Sclerotinia sclerotiorum* during infection of *Glycine max*: on the road to pathogenesis. BMC Genomics 20:157. doi: 10.1186/s12864-019-5517-4, PMID: 30808300 PMC6390599

[ref87] Willbur J. F. Ding S. Marks M. E. Lucas H. Grau C. R. Groves C. L.. (2017). Comprehensive sclerotinia stem rot screening of soybean germplasm requires multiple isolates of *Sclerotinia sclerotiorum*. Plant Dis. 101, 344–353. doi: 10.1094/PDIS-07-16-1055-RE, PMID: 30681926

[ref88] Winton L. M. Krohn A. L. Leiner R. H. (2006). Genetic diversity of sclerotinia species from Alaskan vegetable crops. Can. J. Plant Pathol. 28, 426–434. doi: 10.1080/07060660609507316

[ref89] Wu B. M. Subbarao K. V. (2006). Analyses of lettuce drop incidence and population structure of *Sclerotinia sclerotiorum* and *S. minor*. Phytopathology 96, 1322–1329. doi: 10.1094/PHYTO-96-1322, PMID: 18943664

[ref90] Xu L. Li G. Jiang D. Chen W. (2018). *Sclerotinia sclerotiorum*: an evaluation of virulence theories. Annu. Rev. Phytopathol. 56, 311–338. doi: 10.1146/annurev-phyto-080417-050052, PMID: 29958073

[ref91] Yu Y. Cai J. Ma L. Huang Z. Wang Y. Fang A.. (2020). Population structure and aggressiveness of *Sclerotinia sclerotiorum* from rapeseed (*Brassica napus*) in Chongqing city. Plant Dis. 104, 1201–1206. doi: 10.1094/PDIS-07-19-1401-RE, PMID: 32065567

[ref92] Yu Y. Xioa J. Du J. Yang Y. Bi C. Qing L. (2016). Disruption of the gene encoding endo-β-1,4-xylanase affects the growth and virulence of *Sclerotinia sclerotiorum*. Front. Microbiol. 7:1787. doi: 10.3389/fmicb.2016.01787, PMID: 27891117 PMC5103160

[ref93] Zubrzycki J. E. Maringolo C. A. Filippi C. V. Quiróz F. J. Nishinakamasu V. Puebla A. F.. (2017). Main and epistatic QTL analyses for sclerotinia head rot resistance in sunflower. PLoS One 12:e0189859. doi: 10.1371/journal.pone.0189859, PMID: 29261806 PMC5738076

