# Supplementary figures and images for: Population and genome-wide association studies of Sclerotinia sclerotiorum isolates collected from diverse host plants throughout the United States
Source: Front Microbiol. 2023 Sep 27;14:1251003. doi: 10.3389/fmicb.2023.1251003 (PMC10566370; doi:10.3389/fmicb.2023.1251003)

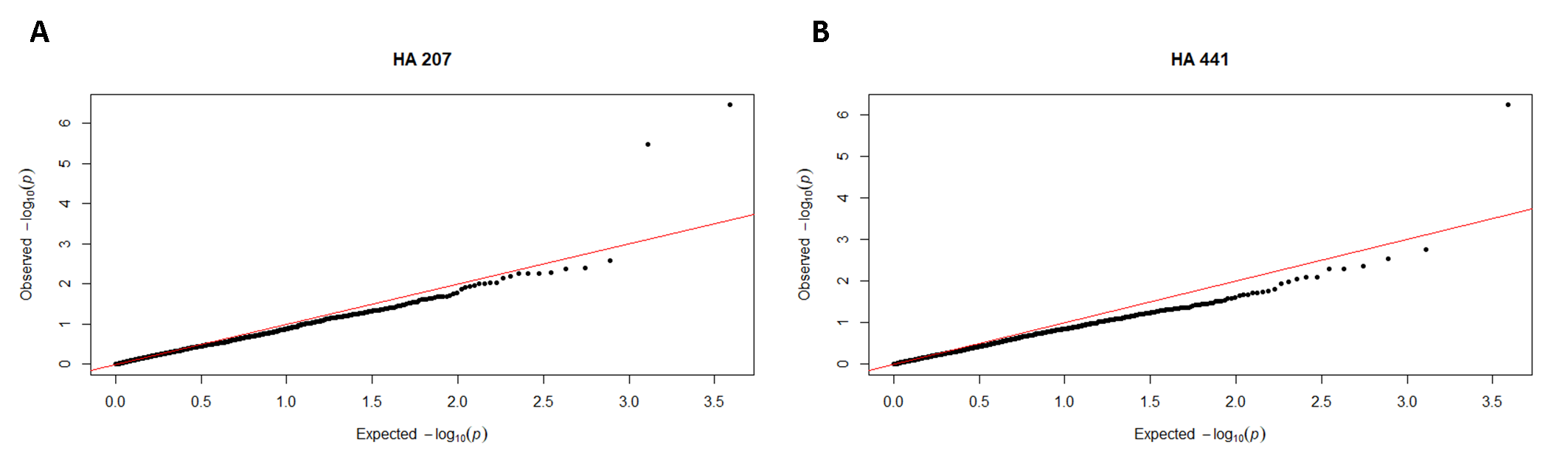

Supplement: Supplementary Figure S1 — Quantile-quantile plots from genome-wide association analyses for aggressiveness of 219 S. sclerotiorum isolates in causing stem lesions on sunflower inbred lines HA 207 (A) or HA 441 (B) using 1937 SNP markers. [file Image_1.TIF]
